# Circulatory support exceeding five years with a continuous-flow left ventricular assist device for advanced heart failure patients

**DOI:** 10.1186/s13019-015-0306-x

**Published:** 2015-08-08

**Authors:** Jan D. Schmitto, Jasmin S. Hanke, Sebastian Rojas, Murat Avsar, Doris Malehsa, Christoph Bara, Martin Strueber, Axel Haverich

**Affiliations:** Department of Cardiac, Thoracic, Transplantation and Vascular Surgery, Mechanical Cardiac Circulatory Support and Cardiac Transplantation Program, Hannover Medical School, OE 6210, Carl-Neuberg-Str, 1, 30625 Hannover, Germany

**Keywords:** Left ventricular assist device, LVAD, HeartMate II, 5-year-follow-up, Long-term support

## Abstract

Continuous-flow left ventricular assist devices (CF-LVAD) are now providing years of safe circulatory support with enhanced quality of life. We present four cases of patients with advanced heart failure who received support for greater than 5-years with the HeartMate II CF-LVAD. One patient continues with support at 7.5 years and has decided to not undergo cardiac transplantation. Another patient has also had LVAD support for 7.7 years, initially with a pulsatile flow LVAD, and then was switched to the HeartMate II, which has continued to support him for the last 6.6 years. Two other patients have undergone heart transplant after support times of 5.46 years and 5.75 years. Few complications occurred and the patients had very active lifestyles during support. Explant analysis revealed very low bearing wear and minimal pannus.

## Background

The durations of support with implantable mechanical circulatory support devices have increased in recent years with the introduction of continuous-flow left ventricular assist devices (CF-LVAD). Safer long-term support has been achieved because of improved device durability and an overall reduction in life-threatening complications [1]. Improved outcomes achieved over the past decade have increased the confidence of physicians and patients for longer durations of CF-LVAD support. [2] Transplant candidates can wait safely for extended durations while their status at transplant is optimized, aiding post-transplant survival. Patients supported for destination therapy who remain free of serious complications can live for many years with active lifestyles and an acceptable quality of life [3]. In this report, we describe four cases in which patients with active lifestyles were supported with CF-LVAD for over 5 years.

## Method

A systematic, retrospective review of patient data was carried out. All patients who were on support by HeartMate 2 for more than five years were considered for this paper. Electronic medical records were reviewed hospital course, for outcome and complications.

## Results

### Case 1

**Fig. 1:**
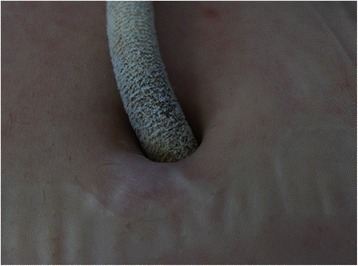
The driveline exit site in patient 1 after 7.5 years of support

A 46-year-old male with ischemic cardiomyopathy was presented with worsening heart failure symptoms and underwent implantation of a HeartMate II CF-LVAD (Thoratec Corporation, Pleasanton, CA, USA) for temporary support until transplant. The postoperative recovery was uneventful, and the patient was discharged from the hospital 23 days after the implant. He resumed an active life style and rehospitalization was only for exchange of a defibrillator battery. Acquired von Willebrand syndrome and hemolysis were observed approximately one year after implant and were treated by a slight reduction in anticoagulation therapy and intravenous Haemate P (coagulation factor VIII). The patient is followed at regular outpatient clinic visits at approximately 2-month intervals. The device pump speed is maintained at 9400 to 9600 RPM and the estimated pump flow is approximately 5.0 L/min. Phenprocoumon (Coumarin) dose is adjusted to achieve an international normalized ratio (INR) of approximately 2.5, and the patient takes 75 mg clopidogrel daily. There have been no problems related to LVAD equipment maintenance. The driveline exit site has been free from infection for the duration of his circulatory support (Fig. 1). This patient has decided to not undergo heart transplant, he exercises at a gym twice a week and works as an office supervisor, and support continues 7.63 years after implantation.

### Case 2

A 56-year-old male with dilated cardiomyopathy presented in October 2005 with NYHA class IV symptoms, pulmonary hypertension, and right heart failure. A pulsatile-flow HeartMate I (XVE-LVAD) was implanted and supported the patient for 13 months until a motor failure, which required the LVAD to be replaced with a HeartMate II device in November 2006. Postoperatively, right heart failure required inotropic support and mechanical ventilation for 10 days. Other postoperative complications included atrial fibrillation, multiple hematomas at the driveline exit site, acquired von Willebrand syndrome, diabetes, knee arthritis and chronic renal insufficiency. The patient left the intensive care unit after 12 days and was discharged from the hospital on postoperative day 49. Since discharge, the patient has required care for ascites, renal insufficiency, and thrombosis of the right jugular vein. The patient is currently receiving dialysis. Although the patient has had multiple complications (unrelated to the device), he has enjoyed an active life during his 7.70 years of mechanical circulatory support, including currently 6.60 years on the HeartMate II CF LVAD. He is retired, and tends to gardening as a hobby.

### Case 3

**Fig. 2:**
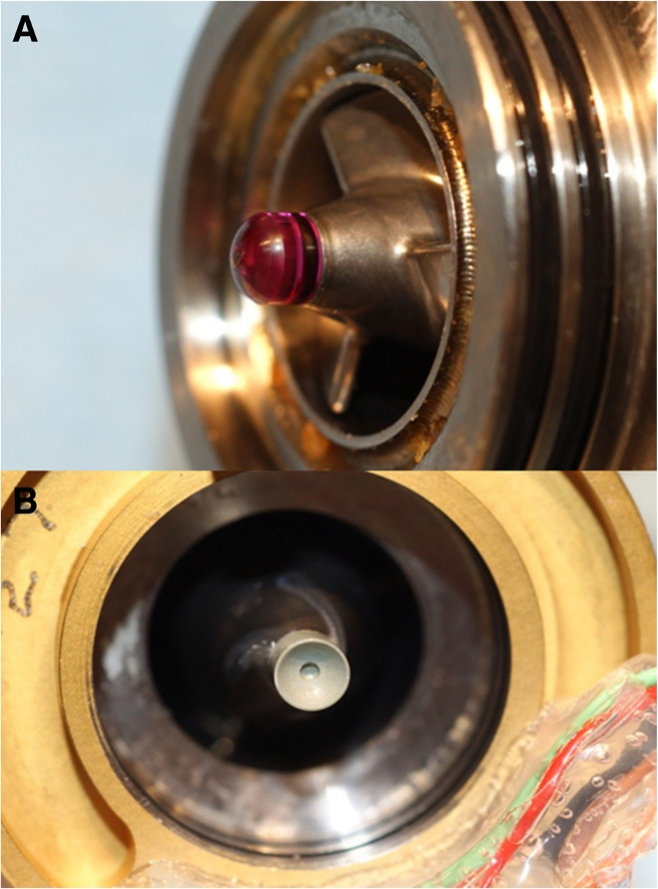
The inlet bearing ball (**a**) and cup (**b**) explanted from the patient in case 3 after 5.46 years of LVAD support are free of accumulated thrombus and demonstrate low bearing wear

**Fig. 3:**
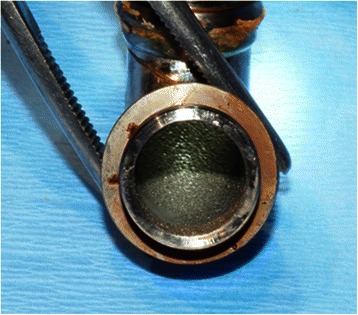
The absence of pannus in the interior of the outflow elbow explanted from the patient in case 3 after 5.46 years of LVAD support

A 23-year-old male diagnosed with dilated cardiomyopathy presented with acute decompensated heart failure and was being treated with high-dose dobutamine. Due to worsening renal and hepatic function, a HeartMate II was implanted as a bridge to transplant. Upon anesthesia induction, the patient experienced a cardiac arrest, which was promptly treated with cardiopulmonary resuscitation (CPR) and then cardiopulmonary bypass. The LVAD was implanted in the usual manner, and a MEDOS HIA VAD (MEDOS Medizintechnik AG, Stolberg, Germany) temporary right ventricular assist device (RVAD) was also implanted due to intermittent right heart failure. Renal failure with dialysis, postoperative bleeding that required surgical reexploration, hepatic dysfunction and cardiac arrhythmias complicated the postoperative course. After 4 days of RVAD support the device was removed. On postoperative day 17, the patient experienced ventricular fibrillation that was treated with defibrillation and implantation of an implantable cardioverter defibrillator (ICD). Recovery was gradual, dialysis and mechanical ventilation were weaned, and the patient was transferred from the intensive care unit on postoperative day 94. After complete rehabilitation, the patient was discharged from the hospital and lived an active life without serious complications. On December 19, 2010, after 5.46 years of LVAD support, the patient underwent successful heart transplant. Analysis of the explanted LVAD (Fig. 2) showed a clean pump with no accumulated thrombus and very low bearing wear of 0.381 μm over 5.46 years (.070 μm/year) consistent with estimated bearing life of well over 17 years of support as previously reported [4]. In addition, the outflow conduit is clean and without pannus formation in the outflow conduit (Fig. 3).

### Case 4

A 21-year-old male with dilated cardiomyopathy experienced acute decompensation that was treated by implantation of a HeartMate II LVAD. Postoperatively, the patient experienced hemorrhagic bronchitis, pneumonia, pulmonary hypertension, gastritis, and hepatitis that eventually resolved with therapy. After complete recovery and rehabilitation, the patient was discharged from the hospital on postoperative day 23. The patient has had elevated lactate dehydrogenase (LDH) in the range of 400 to 1000 IU/L, but clinically significant device thrombosis has not been detected with careful monitoring. The LVAD is maintained at a speed of 9600 RPM, and the average calculated flow rate is 4.6 L/min. The patient had a very active lifestyle while on LVAD support including regular participation in sports (cycling, soccer). The patient experienced a fractured humerus that occurred during a sporting activity, which was repaired and has healed. Due to the patient’s high activity level, a wire within the external portion of the driveline fractured and required repair. After 5.75 years of LVAD support, the patient underwent successful heart transplant. He continues to actively participate in sports at a fitness center and plays soccer on a German amateur team. Explant analysis showed the pump free of accumulated thrombus and with low bearing wear of 1.163 um over 5.75 years (.202 um/yr.), consistent with findings from case 2.

## Discussion

These cases demonstrate that safe long-term mechanical circulatory support exceeding five years is achievable with today’s technology. This provides options that were not available a few years ago for advanced heart failure patients where heart transplantation is not possible or desirable. The HeartMate II axial-flow LVAD with a single moving component, the impeller, has been demonstrated to be very durable in more than 14,000 implants worldwide. In our experience, the HeartMate II is an extremely reliable LVAD system, which has been demonstrated by our stable and low incidence of pump thrombosis over many years [5].

Extended support exceeding 5 years with the HeartMate II has not been previously described in scientific publications, but there are over 120 patients being supported for over 5 years with the longest now reaching over 8 years according to the company’s registry. There have been no reports of mechanical pump bearing or motor failure with the HeartMate II.

On the basis of our experience, the key to successful long-term destination therapy is a structured outpatient clinic, which tremendously helps to avoid life-threatening complications. Careful monitoring of anticoagulation therapy is essential to avoid thrombotic and hemorrhagic complications. Meticulous driveline care and stabilization minimizes infectious problems. ICD implantation and routine monitoring of electrolytes with treatment control arrhythmias. Complete rehabilitation, good nutrition, and exercise aid in avoiding complications. Finally, patient monitoring for problems and an appropriate response plan aids in prompt resolution of problems.

## Conclusion

The HeartMate II CF-LVAD has excellent clinical durability for over 5 years to provide long-term hemodynamic support options for patients with advanced heart failure. Comprehensive patient education, vigilant outpatient care and thorough monitoring to avoid adverse events are important contributions to safe long-term support.
